# Detection of Marker Associated with CTC in Colorectal Cancer in Mononuclear Cells of Patients with Benign Inflammatory Intestinal Diseases

**DOI:** 10.3390/cancers14010047

**Published:** 2021-12-23

**Authors:** Johanna Born, Alexander Hendricks, Charlotte Hauser, Jan-Hendrik Egberts, Thomas Becker, Christian Röder, Susanne Sebens

**Affiliations:** 1 Institute for Experimental Cancer Research, Kiel University and University Hospital Schleswig-Holstein Campus Kiel, Arnold-Heller-Str. 3, Building U30 Entrance 1, 24105 Kiel, Germany; stu127932@mail.uni-kiel.de (J.B.); c.roeder@email.uni-kiel.de (C.R.); 2 Department of General, Visceral, Thoracic, Transplantation and Pediatric Surgery, University Hospital Schleswig-Holstein Campus Kiel, Arnold-Heller-Str. 3, Building C, 24105 Kiel, Germany; alexander.hendricks@med.uni-rostock.de (A.H.); charlotte.hauser@uksh.de (C.H.); j.egberts@ik-h.de (J.-H.E.); thomas.becker@uksh.de (T.B.)

**Keywords:** liquid biopsy, circulating tumor cells, colorectal cancer, chronic intestinal inflammation, keratin-20

## Abstract

**Simple Summary:**

Colorectal carcinoma (CRC) is one of the most frequent cancers in western countries, and non-invasive methods for early detection are still needed. Circulating tumor cells (CTC) in blood of CRC patients have been proven as prognostic and predictive biomarker; however, the suitability of CTC-associated markers for early CRC detection and discrimination from benign diseases has not been analyzed. Thus, this study investigated whether CTC-associated markers can also be detected in the blood of patients with benign inflammatory intestinal disease (IID) or whether they are specific for malignancy. The detection rate of CK20 and DEFA5 clearly differed in diseased patients and healthy controls, while LAD1 and PLS3 was found in all samples but with clear qualitative differences in gene expression. No association between inflammation severity and CTC marker expression was found in IID patients. Finally, PLS3 was identified to be a suitable marker for differentiation between malignant and non-malignant intestinal diseases or healthy controls.

**Abstract:**

Colorectal carcinoma (CRC) belongs to the most common tumor entities in western countries. Circulating tumor cells (CTC) in blood of CRC patients are a powerful prognostic and predictive biomarker. However, whether CTC-associated markers can also be used for early CRC detection and discrimination from benign diseases is not known. This study investigated the presence of CTC-associated markers CK20, PLS3, LAD1, and DEFA5 in blood of patients with benign inflammatory intestinal disease (IID) and their correlation with malignancy. The detection rate of CK20 and DEFA5 significantly differed between diseased patients and healthy controls. LAD1 and PLS3 were detected in all samples with clear differences in gene expression. DEFA5 expression was higher in CRC and IID patients compared to healthy donors, while CK20 and PLS3 were lower in CRC compared to IID patients or healthy controls. Overall, all CTC-associated markers were detectable in blood of IID patients, but not correlating with inflammation severity. Finally, PLS3 emerged as a suitable marker for differentiation between malignant and non-malignant intestinal diseases or healthy controls, however its suitability for early CRC detection needs to be further validated.

## 1. Introduction

Inflammatory bowel disease (IBD) is considered being the result of a non-infectious chronic inflammation of the gastrointestinal tract, and primarily includes the clinically typical manifestations such as Crohn’s disease (CD) and ulcerative colitis (UC). Over the past decades, the prevalence of IBD has increased substantially—in 2017 there were 6.8 million cases globally [1]. The occurrence of IBD is associated with an increased risk for intestinal cancer such as colorectal cancer (CRC) [2,3,4], most likely due to the ongoing and tenacious inflammation of the intestine [5].

On the other hand colorectal cancer (CRC) remains a major health burden and is expected to increase to more than 1 million deaths per year worldwide by 2030 [6]. In industrialized countries, the instrumental screening for CRC by means of repetitive colonoscopies is well established [7] and aids in significantly decreasing the incidence of late-stage CRC, though the average five-year survival rate still is below 70% [8]. However, this procedure is quite invasive and not all patients (e.g., higher age, comorbidities) are eligible for this procedure. Hence, novel biomarkers allowing early detection of CRC and concomitantly specific discrimination from benign intestinal diseases, and which are easily accessible are urgently needed.

In general, the detection of a biomarker in liquid biopsies, e.g., enumeration and characterization of circulating tumor cells (CTC), represents a powerful und reasonable strategy for early cancer detection as well as proof of minimal residual disease, as we demonstrated previously [9]. 

The concept of detecting CTC and its clinical impact for the prognosis of patients with malignant diseases has long been established [10,11]. Manifold approaches for the analyses of CTC with varying sensitivity and specificity rates as well as diverse technological strategies have been introduced over the last decades. In general, two fundamental concepts can be differentiated—(i) the molecular guided approach which indirectly detects CTC mainly by PCR [12,13,14,15,16], and (ii) an immunostaining cytological approach for direct CTC detection and quantification [17,18,19,20]. 

Mostert et al. combined enrichment of CTC using the CellSearch immunological-cytological approach with a subsequent RT-PCR-based molecular characterization of CTC in metastatic CRC patients compared to healthy donors. This study revealed a subgroup of patients in which no CTC were detectable using the CellSearch system but which exhibited a CTC-specific gene panel which might be helpful for therapy decision making [21]. By using a highly specific and sensitive RT-qPCR approach for detection of cytokeratin 20 (CK20) expression in mononuclear cells (MNC) from peripheral blood of CRC patients, we could already demonstrate that CK20 is an independent negative prognostic marker in CRC patients. It was also shown that CK20 is a marker for response after neoadjuvant chemoradiation but not prognosis in patients with rectal cancer [12,13,14]. Furthermore, our recent study indicates that this molecular approach detecting CK20 expression in blood samples of CRC patients might also be a valuable tool for early detection of relapses [9]. Based on this finding it can be speculated that CK20 might also be suitable for early CRC detection.

Importantly, as etymologically specified by the designation of the term CTC, these cells usually are thought to be in conjunction with a malignant disease. However, the markers used to detect CTC are not carcinoma-specific as they identify epithelial cells in general, e.g., cytokeratins or epithelial cell adhesion molecule (EpCAM). Thus, CK20 is found in mature enterocytes but also commonly in CRC cells [22]. Of note, all approaches focusing on epithelial markers for CTC detection are potentially vulnerable because cancer cells may undergo epithelial–mesenchymal transition (EMT) which is characterized by downregulation of epithelial markers, including cytokeratins and EpCAM, leading to dissemination of tumor cells away from the primary tumor into the circulation [23,24]. Therefore, the implementation of other markers for CTC detection which are independent of EMT is reasonable in order to increase CTC detection rates. 

To consider this issue, we implemented Plastin-3 (PLS3), Ladinin-1 (LAD1), and Defensin alpha 5 (DEFA5) as further markers besides CK20 to molecularly assess CTC count in our study.

PLS3 has been identified as a suitable and prognostic marker for CTC detection in metastatic CRC, being also expressed in CTC which have undergone EMT [25,26]. Besides, PLS3 has been proven to be a prognostic and/or diagnostic tumor marker also in other tumor entities, e.g., non-small-cell lung cancer (NSCLC) or pancreatic ductal adenocarcinoma (PDAC) [27,28]. 

Expression of the anchoring filament protein LAD1 has been shown particularly for epithelial organs, with the highest levels in the skin and gastrointestinal tract (GeneCards^®^), qualifying it as a marker for intestinal epithelial/CRC cells, similar to CK20. Accordingly, Moon et al. detected elevated LAD1 expression in CRC tissues correlating with poor prognosis and LAD1 expression was higher in metastatic tissues compared to primary CRC tissues. In line, they also showed that LAD1 promotes migration and invasion of CRC cells [29], further supporting the suitability of this marker for detection of CTC in CRC patients. 

Defensins belong to the antimicrobial cytotoxic peptides and are part of the non-specific immune defense. They are expressed in the surface epithelium of various organs, especially of the small and large intestine [30]. DEFA5 is mainly known for its high expression in the secretory Paneth cells of the terminal ileum (Genecards^®^). DEFA5 overexpression has been demonstrated in UC and CD patients compared to healthy controls [31,32,33,34]. Nastase et al. suggested DEFA5 as a prognostic and predictive biomarker even for early CRC. They demonstrated increased DEFA5 expression especially in colon adenomas indicating that it plays a role in tumorigenesis via the adenoma-carcinoma sequence [35]. Based on these findings it can be speculated whether DEFA5 is a suitable marker for discrimination between benign and malignant intestinal diseases.

To date, only very few investigations have been performed exploring the clinical value of CTC and CTC-associated markers in patients with benign intestinal inflammatory diseases (IID) and particular patients suffering from IBD. The present study aimed at detecting CTC- and malignancy-associated markers in CRC and IID patients as well as healthy donors, and to test whether these markers are specific for colorectal malignancies. For this purpose, CK20, PLS3, LAD1, and DEFA5 were comparatively analyzed by RT-PCR in MNC from blood samples of 98 CRC and 64 IID patients as well as of 40 healthy donors.

## 2. Results

### 2.1. Patients and Demographics

As listed in Table 1, 98 patients with a histopathologically confirmed CRC, 64 patients with IID, and 40 healthy donors were analyzed in this study. The cohort of CRC patients comprised 41 female and 57 male patients with a mean age of 69 years. Seven patients were diagnosed with a UICC stage I cancer, 43 patients with UICC II, 6 patients with UICC III, and 42 patients with UICC stage IV. In the cohort of IID patients, 34 female and 30 male patients with a mean age of 48 years were included, 26 patients suffering from Crohn’s disease (CD), 22 from ulcerative colitis (UC), 2 from indeterminate colitis, and 14 from a sigmoid diverticulitis. The cohort of healthy donors comprised 25 female and 15 male donors with a mean age of 40 years. 

### 2.2. Variable Positivity of CK20 and DEFA5 in Blood Samples of CRC and IID Patients as Well as Healthy Controls

**Figure 1:**
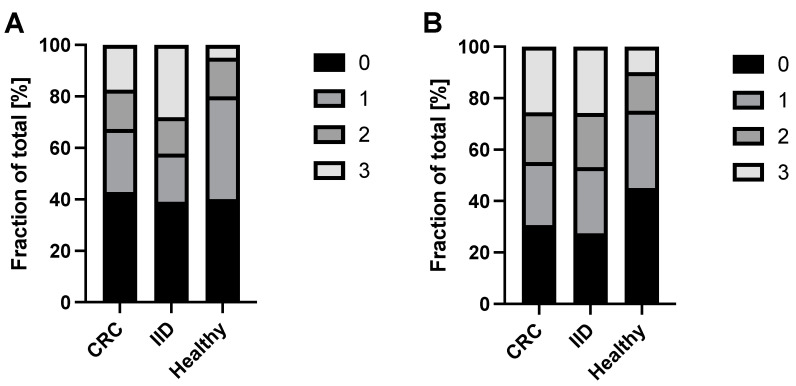
Detection rate of CK20 and DEFA5 in blood samples of CRC and IID patients as well as healthy controls. MNC-derived cDNA from *n* = 98 CRC patients, *n* = 64 IID patients and *n* = 40 healthy controls was analyzed by RT-qPCR for expression of (**A**) CK20 and (**B**) DEFA5. Data are presented as % of samples with no (0), one (1), two (2), or all (3) positive triplicates in the RT-qPCR.

After having performed RT-qPCR for CK20, DEFA5, PLS3, and LAD1 with mononuclear cell (MNC)-derived cDNA, we first analyzed the positivity of either marker in the blood samples of each cohort. In general, in the case of higher marker expression (lower Ct values), all replicates were positive, while in the case of border-line marker expression (higher Ct values), less replicates were positive. Accordingly, PLS3 and LAD1 expression were detectable in all three technical replicates of each individual tested, irrespective of the health status (Table 2), while clear differences were found for the detection rates of CK20 and DEFA5 (Table 2, Figure 1). 

For CK20, the percentage of triple negative samples was comparable in healthy donors (40%), IID (39%) and CRC (43%) patients. However, healthy donors exhibited the lowest amount of triple positive samples (5%), reflecting basal/background expression in healthy individuals, compared to IID (28%) and CRC (17%) patients (Table 2, Figure 1A). Vice versa, IID patients (42%) and CRC (32%) patients presented a notably higher positivity rate (with double and triple positive samples) than healthy donors (20%), albeit IID patients exhibited an even higher positivity rate (42%) than CRC (32%) patients.

For DEFA5, the percentage of triple negative samples was highest in the healthy donors (45%), whereas lower and at a comparable level in IID (27%) and CRC (31%) patients. In contrast, healthy donors exhibited the lowest amount of triple positive samples (10%) compared to IID (26%) and CRC (26%) patients (Table 2, Figure 1B). Thus, IID and CRC patients revealed a higher positivity by trend compared to healthy donors, but no differences between the two disease groups could be determined. 

In summary, these data indicate that the positivity rate of either marker does not allow a clear discrimination between malignant and benign intestinal diseases.

### 2.3. Variable Expression Levels of CTC-Associated Markers in Blood Samples of CRC and IID Patients as well as Healthy Controls

**Figure 2:**
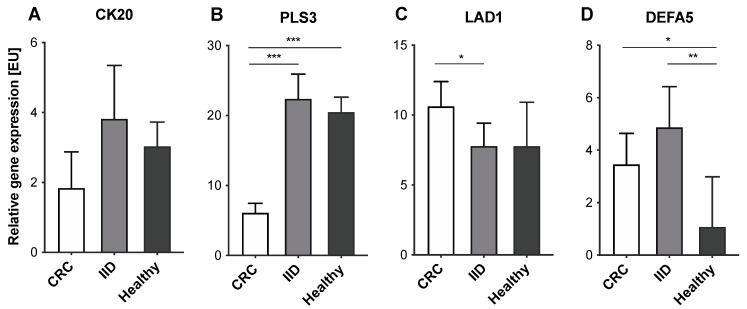
Expression levels of CTC-associated markers in blood samples of CRC and IID patients as well as healthy controls. MNC-derived cDNA from *n* = 98 CRC patients, *n* = 64 IID patients and *n* = 40 healthy controls was analyzed by RT-qPCR for expression of (**A**) CK20, (**B**) PLS3, (**C**) LAD1, and (**D**) DEFA5. Data are presented as median and at a 95% confidence interval. * *p* < 0.05; ** *p* < 0.01; *** *p* < 0.001.

Next, we evaluated the expression levels of either marker in blood samples of CRC and IID patients as well as healthy controls. Regarding CK20, slightly lower expression levels were detected in CRC patients compared to IID patients and healthy controls (CRC—median: 1.84 [EU], range: 0–118.00 [EU]; IID—median: 3.82 [EU], range: 0–97.50 [EU]; healthy—median: 3.03 [EU], range: 0—15.20 [EU]), but no clear differences between the three cohorts could be determined (all *p* = ns) (Figure 2A). PLS3 was detectable at significantly lower expression levels in CRC patients compared to IID patients and healthy controls, the latter two groups showing comparable high expression levels (CRC—median: 6.11 [EU], range: 0–34.19 [EU]; IID—median: 22.37 [EU], range: 3.19–179.10 [EU]; healthy—median: 20.48 [EU], range: 7.84–36.59 [EU]; CRC vs. IID *p* < 0.001; CRC vs. healthy *p* < 0.001; IID vs. healthy *p* = ns) (Figure 2B). Interestingly, LAD1 expression levels were highest in the blood of CRC patients compared to IID patients and healthy controls (CRC—median: 10.62 [EU], range: 0.86–592.70 [EU]; IID—median: 7.79 [EU], range: 0.30–85.91 [EU]; healthy—median: 7.78 [EU], range: 2.90–90.18 [EU]; CRC vs. IID *p* = 0.015; CRC vs. healthy *p* = ns; IID vs. healthy *p* = ns) (Figure 2C). Finally, DEFA5 was detected at significantly higher expression levels in the blood of CRC and IID patients compared to healthy controls; however, there was no clear apparent difference between the two disease groups (CRC—median: 3.46 [EU], range: 0–138.00 [EU]; IID—median: 4.87 [EU], range: 0–47.80 [EU]; healthy—median: 1.08 [EU], range: 0–13.47 [EU]; CRC vs. IID *p* = ns; CRC vs. healthy *p* = 0.023; IID vs. healthy *p* = 0.003) (Figure 2D).

**Figure 3:**
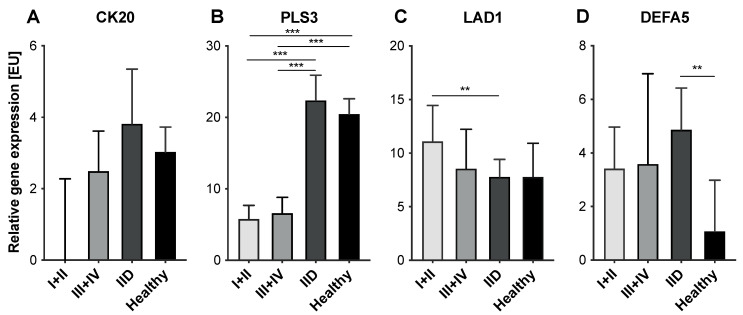
Expression levels of CTC-associated markers in blood samples of CRC patients with different tumor stages, IID patients and healthy controls. MNC-derived cDNA from *n* = 98 CRC patients, *n* = 64 IID patients and *n* = 40 healthy controls was analyzed by RT-qPCR for expression of (**A**) CK20, (**B**) PLS3, (**C**) LAD1, and (**D**) DEFA5. CRC patients were divided in patients with early-stage cancer (UICC I/II) and late-stage cancer (UICC III/IV). Data are presented as median and 95% confidence interval. ** *p* < 0.01; *** *p* < 0.001.

To elucidate whether differences in expression levels of either marker could be determined between healthy controls and IID patients regarding the disease burden of CRC patients expressed by the means of the UICC stage, the sub-cohort of CRC patients was further partitioned and early (UICC I+II) and late-stage cancer patients (UICC III+IV) were stratified. Expression levels of CK20 were higher by trend in patients with late-stage tumor burden (median: 2.49 [EU], range: 0–118.00 [EU]) compared to patients with less advanced disease (median: 0 [EU], range: 0–22.90 [EU]), however, not allowing a clear discrimination from IID patients (all *p* = ns) (Figure 3A). Tumor stage did not show a correlation with the expression levels of PLS3 (stage I+II—median: 5.78 [EU], range: 0.42–34.19 [EU]; stage III+IV—median: 6.58 [EU], range: 0–24.41 [EU]; *p* = ns) indicating that the differing PLS3 expression levels allow discrimination early CRC patients from IID patients and healthy donors (Figure 3B). LAD1 expression was higher in patients with early-stage cancer compared to late-stage cancer (stage I+II—median: 11.10 [EU], range: 0.86–592.70 [EU]; stage III+IV—median: 8.55 [EU], range: 1.77–268.90 [EU]), though no statistical significance could be shown (*p* = ns) (Figure 3C). Hence, these data indicate that the significantly higher LAD1 expression in CRC patients compared to IID patients seemed to be due to an elevated LAD1 expression in early-stage CRC, since patients with UICC stage III+IV presented LAD1 expression levels comparable to IID patients and healthy controls. For DEFA5, the expression levels in early-stage cancer patients (median: 3.41 [EU], range: 0–138.00 [EU]) were comparable to patients with late-stage cancer (median: 3.57 [EU], range: 0–61.73 [EU]) (*p* = ns) and thus were comparable to those of IID patients (Figure 3D).

Overall, these data suggest PLS3 as the only marker allowing a distinct discrimination between healthy individuals, IID patients, and CRC patients, even at early stages. 

### 2.4. Expression Levels of CTC-Associated Markers in Blood Samples of IID Patients Do Not Correlate with Severity of Inflammation

**Figure 4:**
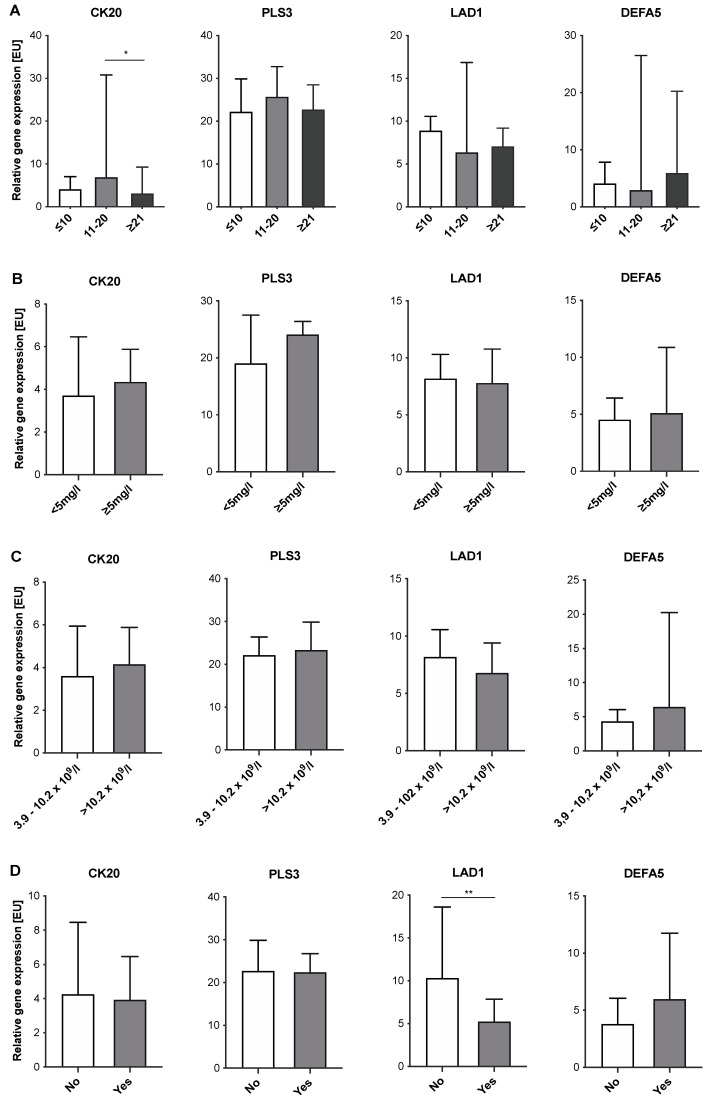
Correlation of expression levels of CTC-associated markers in blood samples of IID patients with clinical parameters of severity of inflammation. MNC-derived cDNA from *n* = 48 IBD patients (for A + D) and *n* = 64 IID (for B + C) was analyzed by RT-qPCR for expression of CK20, PLS3, LAD1, and DEFA5. Expression data were correlated with (**A**) duration of disease [years], (**B**) CRP serum concentration [mg/L], (**C**) blood leucocyte count [cells/L] and (**D**) intake of immunosuppressive drugs [yes/no]. Data are presented as median and 95% confidence interval. * *p* < 0.05; ** *p* < 0.01.

Next, we investigated whether expression levels of the four CTC-associated markers tested correlated with severity of inflammation in IID patients. For this purpose, expression levels of CK20, DEFA5, PLS3, and LAD1 in blood samples of IBD patients were correlated with disease duration (Figure 4A) and the intake of immunosuppressive drugs (Figure 4D). Furthermore, expression levels of all IID patients were correlated with CRP concentrations (Figure 4B) or leucocyte count (Figure 4C). Only the detection of CK20 showed a significant correlation with the disease duration. At a medium disease duration of 11–20 years, expression levels were significantly higher compared to patients with a disease duration of more than 21 years (Figure 4A). Regarding CRP concentrations (Figure 4B) and leucocyte count (Figure 4C), no correlation with expression levels of either marker could be determined. Finally, no clear differences could be observed with respect to the intake of immunosuppressive drugs, despite LAD1 (Figure 4D). Here, intake of immunosuppressive drugs was associated with lower expression levels of LAD1 compared to IBD patients without immunosuppression (Figure 4D). Overall, these data indicate that severity of inflammation does not affect expression levels of CTC-associated markers in the blood of IID patients. However, immunosuppression seems to correlate with a lowered expression of LAD1 in IBD patients.

### 2.5. Suitability of a CTC-Associated Marker for Discrimination of Malignant versus Benign Instestinal Disease

To further validate the suitability of either marker tested for discrimination of particularly malignant and benign colorectal diseases, the expression levels of CK20, PLS3, LAD1, and DEFA5 for healthy controls, IID and CRC patients were subjected to ROC curve analysis for estimation of the discriminatory power between the variables. Based on the previous results for DEFA5, this marker displayed the most significant discrimination between diseased (malignant and benign intestinal diseases) and healthy individuals. Then, the Youden index was determined as the point with the best sensitivity and specificity, from which the specific cut-off values were calculated for each marker.

**Figure 5:**
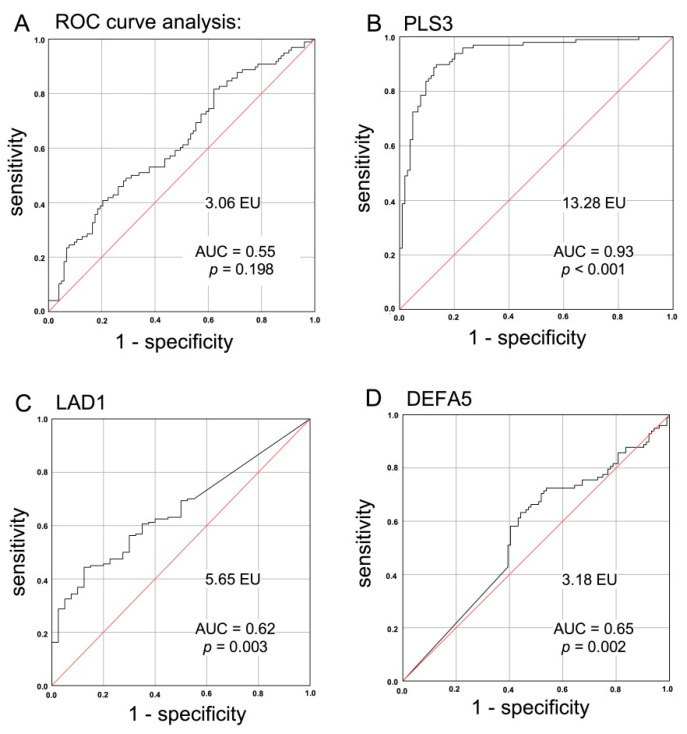
ROC curve analysis of CTC-associated markers for validation of the suitability of either marker for discrimination of malignant and benign colorectal diseases. Expression levels of (**A**) CK20, (**B**) PLS3, (**C**) LAD1, and (**D**) DEFA5 in healthy controls, IID patients, and CRC patients were subjected to ROC curve analysis with respect to the discriminatory power between the variables “malignant disease” versus “benign disease” and “healthy”, respectively, with the exception of DEFA5, where the discriminatory categories were chosen to be “diseased” (CRC + IID) versus “healthy”. Using the ROC curve data, the Youden index J was calculated by the equation J = sensitivity + specificity—1 as the point with the best sensitivity and specificity, from which the specific cut-off values in [EU] were calculated that are mentioned in the figure. AUC = area under the curve.

The cut-off value for CK20 expression was determined at 3.06 expression units (EU), which was not suitable to allow discrimination of CRC patients from IID patients and healthy donors with sufficient specificity and sensitivity. This was also illustrated by the low AUC value of 0.55 in the ROC curve as well as the lack of significance (*p* = 0.198) (Figure 5A). In contrast, with a cut-off value of 13.28 and an AUC value for PLS3 > 0.9, CRC patients could be clearly identified from benign intestinal disease patients and healthy donors with almost 90% sensitivity and specificity (*p* < 0.001, Figure 5B). Among the 13 false positives, 6 were CD patients, 1 UC patient, and 6 were healthy donors. In total, 11 CRC patients showed a false negative result. Regarding TNM stage or other factors, no explanatory influences were identified among the collected data (data not shown). LAD1 presented a good sensitivity of 81% at a cut-off value of 5.65 EU, but with an insufficient specificity of 38%. Thus, the AUC value was 0.62 (*p* = 0.003; Figure 5C). For DEFA5, the calculated cut-off value of 3.18 EU and an AUC value of 0.65 did not allow a sufficient differentiation between diseased (malignant and benign intestinal diseases) and healthy individuals (specificity of 70% and sensitivity of 56%, *p* = 0.002; Figure 5D).

Overall, this analysis suggests a reduced PLS3 expression level in peripheral blood as a promising biomarker with a very good specificity and sensitivity for detection of even early CRC.

## 3. Discussion

Despite significant advances in the field of diagnosis and therapeutic efficacy over the last decades, CRC represents the third most common cancer diagnosis in western countries [36]. Screening programs have been established in several countries; however, colonoscopies are highly invasive and tests for blood in stool are often too unspecific [7]. Therefore, diagnostic tools that are non-invasive and thereby also well suited for elderly individuals on the one hand and that are specific and allow discrimination of benign inflammatory diseases of the intestine on the other hand, are of great interest for CRC prevention. In addition, these strategies might also be useful for detection and prevention of CRC relapses [10,37,38]. Thus, in recent years, the detection of biomarkers in liquid biopsies has become more and more important in patients with solid cancers [39]. In this context, we and others could demonstrate the potential of CTC as predictive and prognostic biomarker in CRC patients [9,12,13,14,17]. Continuing these studies, the present study aimed at detecting CTC-associated markers in CRC and IID patients and to validate whether these markers are specific for colorectal malignancies. The RT-PCR-based analysis of CK20, PLS3, LAD1, and DEFA5 expression in blood samples of 98 CRC and 64 IID patients as well as of 40 healthy donors revealed that PLS3 and LAD1 expression were detectable in almost all analyzed blood samples, while distinct differences in the detection rate of CK20 and DEFA5 could be determined in the three sub-cohorts, however, not allowing a more in-depth discrimination between CRC and IID patients. Furthermore, expression levels of these four markers differed in blood samples. Merely the expression levels of PLS3 and LAD1 significantly differed between CRC and IID patients and healthy donors.

Detection of epithelial cell-related gene expression in patients with benign intestinal diseases has been already described in other studies [40,41]. In line with the study of Hardingham et al., which detected CK20 expression in 50% of the blood samples of patients with inflammatory colon diseases [40], our CK20 detection rate was 60% in IID patients. The slight difference could be explained by the different PCR method used in either study, as well as differences in the dimension and diseases of the patient cohort. Using the CellSearch system and also an epithelial immunospot assay, Pantel et al. determined CTC in blood samples of 11.3% and 18.9% of patients with benign colon diseases, respectively [41]. Irrespective of the different detection methods and rates, the determination of epithelial markers in blood samples of IID patients, which are also used for detection of CTC in CRC patients, indicate that inflammatory processes promote the dissemination of intestinal epithelial cells in the circulation as it has been also observed in other benign gastrointestinal diseases [42,43]. This circumstance might also explain a certain abundance of these markers in blood samples of healthy donors presumably not being aware of a smoldering low-grade intestinal inflammation. However, no correlation of expression levels of either marker and parameter of inflammation severity (disease duration, CRP and leucocyte count) could be assessed in IBD patients.

DEFA5 overexpression was shown in UC and CD patients compared to healthy controls [31,32,33,34] as well as in patients with precancerous conditions such as colonic adenomas [35]. Therefore, DEFA5 was proposed as a prognostic and predictive biomarker for early CRC [35]. In line with these findings, we could detect the highest DEFA5 expression in blood samples of IID patients compared to CRC patients and healthy controls. Moreover, CRC patients also exhibited significantly elevated DEFA5 expression levels; however, with no discriminatory differences between early (UICC I+II) and late (UICC III+IV) stage cancer. This might indicate that DEFA5 expression rises during inflammatory processes as well as early stages of colorectal tumorigenesis. Owing to the fact that we have only incomplete data on the presence of neoplastic cells/preneoplasia in the tissues of IID patients, we can only speculate whether increased DEFA5 expression levels in the blood of our IID patients are indicative of an increased risk for CRC.

In their study, Moon et al. demonstrated increased LAD1 expression rates detected by immunohistochemistry of tumor microarrays of CRC tissue that were correlating with a poor prognosis and was further shown to confer a migratory and invasive phenotype to CRC cells [29]. This observation might indicate that LAD1 is involved in dissemination of CRC cells leading to metastasis consistent with a higher LAD1 expression in metastatic tissues compared to primary CRC tissues. Although the LAD1 expression rates were significantly higher in CRC patients (particularly in the early stages UICC I+II) compared to IID patients of our cohort, expression of LAD1 could be detected in all blood samples of the three sub-cohorts matching its abundant expression in epithelial organs. 

Based on gene expression results, an ROC curve analysis revealed that CK20, DEFA5, and LAD1 are not suited as blood-based biomarkers allowing for a reliable and specific discrimination of a CRC from benign colorectal diseases or healthy individuals. 

As PLS3 was shown to be a suitable and prognostic marker for CTC detection in metastatic CRC and to be detectable in CTC that have undergone EMT [25,26], we also included this marker in our workflow. In concordance with the study of Yokobori et al. [25], we could detect considerable PLS3 expression in blood samples of CRC patients. However, in contrast to the above-mentioned study we could also demonstrate (i) PLS3 detection in blood samples of almost all CRC patients (in contrast to 33.6% in the study of Yokobori et al.) and (ii) a surprisingly similar detection rate in IID patients and healthy donors. Moreover, expression levels of PLS3 were significantly lower in CRC patients compared to the other two cohorts. At first glance, our findings appear contradictory to the findings of Yokobori et al. [25]. However, it should be noted that our study determined PLS3 expression at the gene expression level by RT-PCR, whereas Yokobori et al. assessed it by immunofluorescence staining. In addition, we did not correlate PLS3 expression levels in CRC patients with clinicopathological parameters as it was performed by Yokobori et al. and also Kujawaski et al. [25,26]. Instead, our focus was to elaborate whether this marker is suitable for discrimination between malignant and benign disease or healthy donors. 

However, using our RT-PCR based approach significantly differing PLS3 expression levels between patients with malignant and benign colorectal diseases as well as healthy donors could be determined. These findings along with the results of the ROC curve analysis strongly support the suitability of PLS3 as a biomarker with a very good specificity and sensitivity for detection of a CRC, even at early stages. 

## 4. Conclusions

In summary, the present study depicts and proves the feasibility of the application of a liquid biopsy concept by detection of CTC-associated markers in CRC and IID patients. The discriminative value of PLS3 as a biomarker allowing for the differentiation between CRC patients and non-malignant conditions has been demonstrated by generating a cut-off value. However, future investigations with an even more extensive cohort of patients will have to validate our results and may help to further define an optimal biomarker panel for early detection of CRC.

## 5. Material and Methods

### 5.1. Collection and Preparation of Patient/Donor Blood Samples

In total, blood samples from 98 CRC patients, 64 IID patients, and 40 healthy donors were analyzed. All CRC and IID patients underwent surgery at the Department of General, Visceral, Thoracic, Transplantation and Pediatric Surgery, University Hospital Schleswig-Holstein, Campus Kiel during 2004 until 2019 in order to resect the primary tumor or diseased parts of the intestinal tract. A complete overview of the administered antiphlogistic and immunosuppressive medication is given in Table 3. All patients and healthy individuals gave written informed consent to participating in this study. The study was approved by the local ethics committee of the Medical Faculty, Kiel University and the University Hospital Schleswig-Holstein, Campus Kiel (Reference No. A110/99). Classification of the pathological tumor stage was performed at the Department of Pathology, University Hospital Schleswig-Holstein, Campus Kiel, according to the current UICC-classification, and only patients with a histologically proven CRC were included in the study. Clinical data were obtained from the clinical research database of the oncological biobank of the Comprehensive Cancer Center Kiel (BMB-CCC) and data were verified by re-examination of original patient records. Peripheral blood samples were taken from patients shortly prior to surgery from a central venous line. Blood samples from healthy donors were taken from the medial cubital vein. All samples were further processed within 2 h. Approximately 20 mL of blood in lithium heparin Monovettes (Sarstedt, Nümbrecht, Germany) were used and processed by centrifugation through a Ficoll-Hypaque density cushion (GE Healthcare/Merck, Darmstadt, Germany) in accordance with the supplier’s recommendation for enrichment of the MNC fraction. MNC fractions were washed in phosphate-buffered saline (Thermo Fisher Scientific, Darmstadt, Germany) and cells were subsequently lysed for total RNA preparation in RNAPure^TM^ reagent (VWR Peqlab, Darmstadt, Germany) and stored at −80 °C until further usage. Leukocyte blood count and CRP serum levels were assessed during routine clinical diagnostics.

### 5.2. RNA Isolation and Semi-Quantitative RT-qPCR

The semi-quantitative RT-qPCR for indirect detection of CTC has been previously established [14,17]. Total RNA from MNC fractions was isolated after lysis with RNAPure^TM^ reagent (VWR Peqlab) according to the manufacturer’s protocol and cDNA was synthesized by reverse transcription of 3 μg of total RNA (Maxima First Strand cDNA Synthesis Kit, Thermo Fisher Scientific, Darmstadt, Germany). The qPCR was run in total volumes of 20 μL on 96-well plates (Sarstedt, Nümbrecht, Germany), each sample in triplicate. The TaqMan Fast Advanced Master Mix on a StepOne Plus real-time PCR System (all Thermo Fisher Scientific, Darmstadt, Germany) and the following TaqMan-kits were used for the real-time gene expression analysis: Cytokeratin-20/KRT20 (CK20), Hs00966063_m1; DEFA5, Hs00360716_m1, LAD1, Hs00194326_m1; Plastin 3/PLS3, Hs00958350_m1, and the housekeeping gene TBP (TATA-box binding protein), Hs00427620_m1, used as a reference (Thermo Fisher Scientific, Darmstadt, Germany). Relative gene expression was calculated as arbitrary expression units [EU] by the ΔC_t_ method based on the difference between C_t_ of the genes mentioned before and the reference gene TBP-C_t_ values computed using the StepOne software (Thermo Fisher Scientific, Darmstadt, Germany).

### 5.3. Statistical Analysis

Statistical analysis was performed using SPSS^®^ software (IBM Deutschland GmbH, Ehningen, Germany). First, a test for normal distribution was performed using the Shapiro–Wilk test. A t-test was performed on independent samples to analyze the means of two parametric data sets. For non-parametric data sets, a Mann–Whitney U test was performed. Analysis of three or more samples was performed using one-factorial ANOVA (analysis of variance) or, for non-normally distributed data, the Kruskal–Wallis test. Tests for independence between categorical variables were analyzed using cross-tabulations and a chi-square test. A receiver operating characteristic (ROC) curve was used to classify the diagnostic strength of the markers studied to illustrate the trade-off between high sensitivity and high specificity in distinguishing between clinically normal and clinically abnormal laboratory values. The cut-off value was determined using the Youden index.

## Figures and Tables

**Table 1:** Demographics of CRC and IID patients as well as healthy donors along with clinical characteristics of the entire study population.

Parameter	CRC Patients N (%)	IID Patients N (%)	Healthy Donors N (%)
	98 (100)	64 (100)	40 (100)
**Gender**			
Female	41 (42)	34 (53)	25 (62,5)
Male	57 (58)	30 (47)	15 (37,5)
**Age in years**			
20–29	0 (0)	11 (17)	10 (25)
30–39	1 (1)	9 (14)	10 (25)
40–49	5 (5)	13 (20)	8 (20)
50–59	14 (14)	15 (24)	10 (25)
60–69	24 (25)	8 (13)	2 (5)
70–79	40 (41)	6 (9)	0 (0)
>80	14 (14)	2 (3)	0 (0)
mean	69	48	40
**UICC stage**			
I	7 (7)	-	-
II	43 (44)	-	-
III	6 (6)	-	-
IV	42 (43)	-	-
**Inflammatory intestinal disease**			
Crohn’s disease	-	26 (41)	-
Ulcerative colitis	-	22 (34)	-
Indeterminate colitis	-	2 (3)	-
Sigmoid diverticulitis	-	14 (22)	-

Abbreviations: UICC—Union Internationale Contre le Cancer, CRC—colorectal cancer; IID—inflammatory intestinal disease.

**Table 2:** Detection rate of CTC-associated marker sCK20, DEFA5, PLS3, and LAD1 in MNC-derived RNA from blood samples of CRC and IID patients as well as healthy donors.

Parameter	CRC Patients N (%)	IID Patients N (%)	Healthy Donors N (%)	*p*
	98 (100)	64 (100)	40 (100)	
**CK20**				
+	56 (57)	39 (61)	24 (60)	0.880
-	42 (43)	25 (39)	16 (40)	
**CK20 detection**				
0	42 (43)	25 (39)	16 (40)	0.064
1	24 (25)	12 (19)	16(40)	
2	15 (15)	9 (14)	6 (15)	
3	17 (17)	18 (28)	2 (5)	
**DEFA5**				
+	68 (69)	45 (73)	22 (55)	0.154
-	30 (31)	17 (27)	18 (45)	
**DEFA5 detection**				
0	30 (31)	17 (27)	18 (45)	0.340
1	24 (24)	16 (26)	12 (30)	
2	19 (19)	13 (21)	6 (15)	
3	25 (26)	16 (26)	4 (10)	
**PLS3**				
+	97 (99)	64 (100)	40 (100)	1
-	1 (1)	0 (0)	0 (0)	
**LAD1**				
+	98 (100)	63 (100)	40 (100)	1
-	0 (0)	0 (0)	0 (0)	

Abbreviations: CRC—colorectal cancer; IID—inflammatory intestinal disease. + = positive PCR detection, meaning in at least one of three technical replicates marker expression is detectable;—or 0 = negative PCR detection; 1 = one of three technical replicates is positive; 2 = two of three technical replicates are positive; 3 = all technical replicates are positive.

**Table 3:** Overview of the administered antiphlogistic and immunosuppressive medication.

Immunosuppressive Medication	Patient Count N (%)
	36 (100)
**Combination therapy**	
Aminosalicylates + glucocorticoids	9 (25)
Aminosalicylates + purine nucleoside analogues	1 (3)
Aminosalicylates + monoclonal antibodies	4 (11)
Glucocorticoids + purine nucleoside analogues	3 (8)
Glucocorticoids + monoclonal antibodies	10 (28)
Purine nucleoside analogues + monoclonal antibodies	1 (3)
Aminosalicylates + glucocorticoids + monoclonal antibodies	1 (3)
Aminosalicylates + glucocorticoids + purine nucleoside analogues	1 (3)
**Monotherapy**	
Mesalazine	3 (8)
Prednisolone	1 (3)
Adalimumab	1 (3)
Infliximab	1 (3)

Aminosalicylates: Mesalazine, Sulfasalazine; Glucocorticoids: Prednisolone, Hydrocortisone, Budenoside; Purine nucleoside analogues: Azathioprine; Monoclonal antibodies: Vedolizumab, Adalimumab, Infliximab, Golimumab, Ustekinumab.

## Data Availability

Data are contained within the article.

## Abbreviations

CD	Crohn’s disease
CK20	Cytokeratin-20
CRC	Colorectal cancer
CRP	C-Reactive protein
CTC	Circulating tumor cells
DEFA5	Defensin alpha 5
EMT	Epithelial–mesenchymal transition
EpCAM	Epithelial cell adhesion molecule
EU	expression units
IBD	Inflammatory bowel disease
IID	Inflammatory intestinal disease
LAD-1	Ladinin-1
MNC	Mononuclear cells
PLS3	Plastin-3
RT-qPCR	Real-time quantitative polymerase chain reaction
SD	Standard deviation
UC	Ulcerative colitis
UICC	Union Internationale Contre le Cancer
